# Changes in agricultural context and mental health of farmers in different regions of Thailand during the fifth wave of the COVID-19 pandemic

**DOI:** 10.1186/s12889-022-14464-3

**Published:** 2022-11-09

**Authors:** Ratana Sapbamrer, Nalin Sittitoon, Aroon La-up, Nisa Pakvilai, Jiraporn Chittrakul, Wachiranun Sirikul, Amornphat Kitro, Surat Hongsibsong

**Affiliations:** 1 Department of Community Medicine, Faculty of Medicine, Chiang Mai University, 110 Inthavaroros Road, Sri Phum Subdistrict, Muang District, Chiang Mai Province, 50200 Thailand; 2 School of Environmental Health, Institute of Public Health, Suranaree University of Technology, Nakhon Ratchasima Province, 30000 Thailand; 3 Mahidol University, Nakhonsawan Campus, Nakhonsawan, 60130 Thailand; 4 Environmental Science Program, Faculty of Science and Technology, Valaya Alongkorn Rajabhat University Under the Royal, Pathum Thani Province, 13180 Thailand; 5 School of Health Sciences Research, Research Institute for Health Sciences, Chiang Mai University, Chiang Mai Province, 50200 Thailand

**Keywords:** COVID-19, Agriculture, Mental health, Farmer, Stress, Anxiety, Depression

## Abstract

**Background:**

Thailand's agricultural sector is crucial to the country's development and economy. The COVID-19 pandemic caused negative effects on the agricultural context and the mental health of Thai farmers. This study aimed to compare changes in the agricultural context and mental health among farmers in different regions of Thailand during the fifth wave of the COVID-19 pandemic. We also investigated the determinants associated with the mental health of farmers.

**Methods:**

This cross-sectional study was carried out from December 2021 to January 2022 in Thailand, with 1,676 Thai farmers interviewed.

**Results:**

The results found that the Central region found to be the highest impact of increased agrochemical costs (91.0%) and decreased crop yields (92.0%), while the highest in the North and the North-East was found in decreased crop product prices (84.0 and 66.4%). In the context of economic status, the greatest impact in the Central region was found to be in increased household expense (96.3%), while the highest impact in the North, the North-East, and the South was found in decreased household income (91.5, 80.0, and 69.8%, respectively). Regarding mental health, the Central region was found to be the highest prevalence of extremely high stress and severe depression symptoms (18.3 and 23.4%), whereas the South region was found to be the highest prevalence of severe anxiety symptoms (7.4%). Interestingly, the multivariate analysis also found that the agricultural factors associated with mental health were decreased working days, changes in agrochemical type and crop cultivation, decreased crop rotation, increased difficulty in accessing agrochemicals and markets, decreased crop yields, and increased household debt.

**Conclusion:**

The findings of this study are useful for government and relevant organizations to plan and implement supportive measures and improve mental health services to mitigate both short and long-term impacts of the COVID-19 pandemic suit on the regions. Specific measures and facilities should be tailored toward the target regions.

**Supplementary Information:**

The online version contains supplementary material available at 10.1186/s12889-022-14464-3.

## Background

The World Health Organization declared the coronavirus (COVID-19) outbreak a global pandemic on March 11, 2020 [1]. At the beginning of February 2022, the number of confirmed cases of COVID-19 globally was approximately 376 million with a death of 5.6 million, the dominant variant at this time being Omicron. In Thailand, the number of confirmed cases of COVID-19 was approximately 4.63 million with 31,915 deaths [2, 3]. In Thailand the COVID-19 pandemic has officially entered the fifth wave of infection, the number of new cases starting to rise again after the 2022 New Year celebrations [4]. Omicron is more likely to spread than other variants, therefore the Thai government has resumed mandatory public health management measures, including lockdown in epidemic areas, working from home, physical distancing, time limits for restaurants, pubs and alcohol drinking, and antigen test kit (ATK) monitoring [5, 6].

Thailand's agricultural sector is crucial to the country's development and economy. The agricultural sector employs approximately 30% of the total workforce and is the income for 6.4 million households [7]. Agriculture accounted for 8.64% of Thailand's total domestic product (GDP) in 2020, 1.36 trillion Baht [8]. The COVID-19 pandemic caused negative effects on agricultural production and food supply security. The pandemic led to a shortage of laborers, import and export restrictions, and transportation restrictions, the economic impact on farm households resulting in increased debt, lower savings, and income loss [9, 10]. Farm households lost 39% of their income due to COVID-19, while overall family income dropped by 16% [7]. As a result, farmers are highly vulnerable to the negative consequences of the COVID-19 crisis.

The COVID-19 pandemic has had an impact on the mental health of the general population and other groups. Experiences in quarantine, unemployment, medical symptoms similar to those of COVID-19 infection, and preventative measures can all contribute to stress, sadness, and anxiety [11]. Previous studies in Thailand also found mental health problems due to the COVID-19 pandemic [12, 13]. A meta-analysis by Nochaiwong et al. [14] stated that the prevalence of mental health problems worldwide in the general population owing to COVID-19 was 36.5% for stress, 28% for depression, 26.9% for anxiety, and 50% for psychological distress, issues also occurring in the farming population. Changes in agricultural production and distribution, as well as family debt, were linked to increased stress and depression levels among farmers [9, 15]. Organization and social support were factors linked to anxiety levels, cropland size, and financial status. As most farmers had low income and lived in rural regions, they were designated as a population vulnerable to the adverse psychological impact of COVID-19 [16, 17]. In addition, most farmers are acknowledged as being informal sector employees with limited social and economic security when compared to other populations [18].

The aim of the study was to compare changes in agricultural context and mental health among farmers in different regions of Thailand. We also investigated the determinants of stress, anxiety, and depression due to changes in agricultural context during the fifth wave of the COVID-19 pandemic. The findings of this study will be useful for government and relevant organizations to provide supportive measures and facilities suit on the regions, with the aim of improving the mental health of farmers.

## Methods

### Settings and participants

A cross-sectional study was conducted from December 2021 to January 2022 during the fifth wave of the COVID-19 in Thailand. Healthy farmers aged ≥ 18 years who lived in four regions of Thailand were invited to participate in the study. Purposive sampling was used for sampling. Ayutthaya, Chiang Mai, Nakorn Ratchasima, and Pattani were chosen as representative areas of the Center, North, North-East, and South regions because these four provinces were agricultural crop areas with a high incidence of cumulative cases of the COVID-19. Three to four agricultural subdistricts in each region were randomly chosen as study sites.

**Fig. 1:**
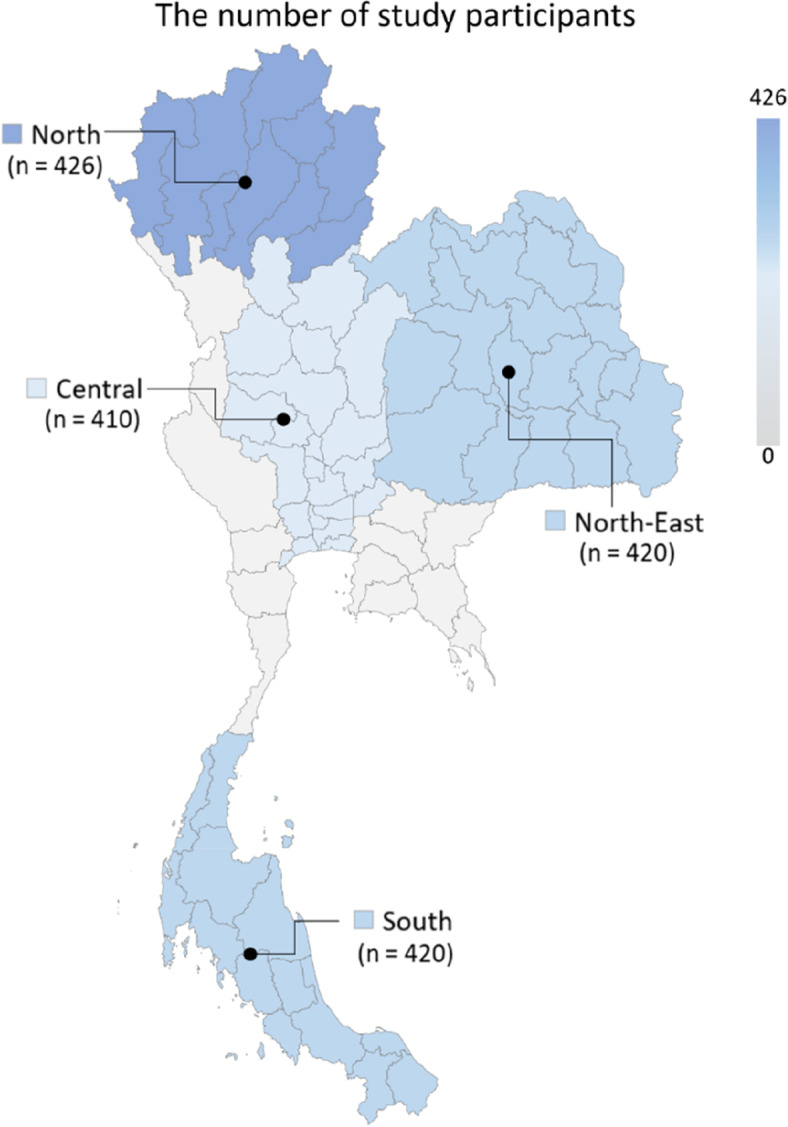
The number of study participants

The sample size was calculated using EpiInfo. The total of the agricultural population in Thailand was 7,271,759, and the prevalence of global mental health problems during the COVID-19 pandemic was within the range of 28–50% [14, 19]. The confidence interval was set at 99.99% and an expected prevalence of 50% was used for calculation. Therefore, the minimum sample size was 1,514. A total of 1,676 farmers were interviewed which included 410 farmers from the Central, 426 from the North, 420 from the North-East, and 420 from the South (Fig. 1).

### Interviews

The majority of farmers usually have limited access to the internet and many are illiterate therefore an in-person interview was used as the research tool. Twelve interviewers (3 interviewers in each region) were trained by the research team before interviewing the participants. The topics of training were as follows: the study’s objectives; components of questions in interview form; characteristics of questions and answers in interview form; interviewer performance; and practice with research team before interviewing the participants.

Because there are regional languages in Thailand, interviewers were recruited based on their home regions. Additionally, recruitment of local interviewers made it easy to travel for interview. Interviewers made an appointment date and time with the participants before entering the study area. The participants who met the inclusion criteria were invited to participate the study. Written informed consent was obtained from the participants before the interview. To reduce the risk of the spread of COVID-19, interviewers and participants wore face masks during the interview. The participants were interviewed for 10–15 min. The interview form included questions on sociodemographic characteristics, changes in agricultural context, and mental health during the fifth wave of the COVID-19 pandemic.

Sociodemographic data included age, gender, marital status, monthly income, education level, region of residence, comorbidity, COVID-19 vaccination, wearing of a face mask, physical distancing, cleaning hands with alcohol, and relationships with people around them during the COVID-19 pandemic. Changes in the agricultural context were divided into 3 categories: crop cultivation, crop products, and agricultural economic status. The questions in the crop cultivation context covered the type of crop cultivation, rotation, duration, method, agricultural working days, laborers, crop protection, costs of crop cultivation, access to agrochemicals, the quantity of agrochemicals, the type of agrochemicals, and cost of agrochemicals. The questions in the crop products context consisted of crop yields, prices of crop products, markets, and logistics. The questions in the economic context consisted of household income, household expense, household debt, and earning extra income. To ensure that the findings of changes in the agricultural context were caused by the fifth wave of the COVID-19 pandemic, the response options in agricultural context questions were typically answered in the direction of changes compared to the period prior to the fifth wave of the COVID-19 pandemic, such as increase direction, and decrease direction.

Mental health was categorized as stress, anxiety, and depression during the fifth wave of the COVID-19 in Thailand. To ensure that the findings of mental health symptoms were caused by the fifth wave of the COVID-19 pandemic, the participants were asked mental health symptoms in the past one month. Stress levels were measured using a 5-point Likert scale, including no stress, mild, moderate, high, and extremely high. The generalized Anxiety Disorder-7 (GAD-7) was used for measuring anxiety [20]. This consisted of 7 items, each item having a 4-point Likert scale, including: not at all, several days, more than half the days, and nearly everyday. The total summative score ranges from 0 to 21. The total summative scores of 0–4, 4–9, 10–14, and 15–21 were used to represent cut off points for minimal, mild, moderate, and severe anxiety, respectively. The GAD-7 has been validated for the measurement of anxiety in the general population [21]. The patient Health Questionnaire (PHQ-9) Thai version was used for measuring depression. The Thai version of the PHQ-9 was translated by Lotrakul et al. [22]. It consists of 9 items, each item having a 4-point Likert scale, including: not at all, several days, more than half of the days, and nearly everyday. The total summative score ranges from 0 to 27. The total summative scores of 0–6, 7–12, 13–18, and 19–21 were used to represent the cut off points for no depression, mild, moderate, and severe depression, respectively. A reliability check refected acceptable internal consistency (Conbach’s alpha = 0.981for stress, 0.916 for GAD-7, and 0.925 for PHQ-9). Theoretical framework of this study is shown in supplementary (Figure S1).

### Statistical analysis

Sociodemographic characteristics, changes in agricultural context, and the mental health of farmers during the fifth wave of the COVID-19 pandemic are presented as frequency and percentage.Univariate analysis (Chi-square test) was used to analyze the crude association between mental health with sociodemographic characteristics and agricultural context. The variables which had significant associations (*P* value < 0.05) with mental health were included in the model of multivariate analysis. Binary logistic regression analysis (Enter model) was used to analyze the determinants of mental health of Thai farmers due to changes in agricultural context during the fifth wave of the COVID-19 pandemic.

## Results

### Sociodemographic characteristics of Thai farmers

Table 1 presents the sociodemographic characteristics of Thai farmers classified by region of Thailand. Approximately half of the farmers were female, over 45 years old, with a monthly income of less than or equal to 5,000 Thai Baht and had been educated to primary level. About 66.8% of the farmers were married, and 72.7% had no comorbidities. Most farmers (94.3%) had had the COVID-19 vaccination.

**Table 1:** Sociodemographic characteristic of Thai farmers

Parameter		n (%)				
		**Total (*n*=1676)**	**Central (n=410)**	**North (n=426)**	**South (*n*=420)**	**North-East (n=420)**
Gender	Male	807 (48.2)	139 (33.9)	294 (69.0)	190 (45.2)	184 (43.8)
	Female	869 (51.8)	271 (66.1)	132 (31.0)	230 (54.8)	236 (56.2)
Age	≤ 45 years	825 (49.2)	370 (90.2)	123 (29.3)	268 (63.8)	123 (29.3)
	> 45 years	851 (50.8)	40 (9.8)	362 (85.0)	158 (36.2)	297 (70.7)
Marital status	Single/divorced	557 (33.2)	183 (44.6)	101 (23.7)	134 (31.9)	139 (33.1)
	Married	1,119 (66.8)	227 (55.4)	325 (76.3)	286 (68.1)	281 (66.9)
Monthly income	THB ≤ 5,000	867 (51.7)	122 (29.8)	316 (74.2)	244 (58.1)	185 (44.0)
	THB > 5,000	809 (48.3)	288 (70.2)	110 (25.8)	176 (41.9)	235 (56.0)
Education level	≤ Primary education	770 (45.9)	69 (16.8)	291 (68.3)	183 (43.6)	227 (54.0)
	≥ Secondary education	906 (54.1)	341(83.2)	135 (31.7)	237 (56.4)	193 (46.0)
Comorbidity	Yes	457 (27.3)	134 (32.7)	139 (32.6)	71 (16.9)	113 (26.9)
	No	1,219 (72.7)	276 (67.3)	287 (67.4)	349 (83.1)	307 (73.1)
COVID-19 vaccination	Yes	1,580 (94.3)	408 (99.5)	396 (93.0)	386 (91.9)	390 (92.9)
	No	96 (5.7)	2 (0.5)	30 (7.0)	34 (8.1)	30 (7.1)
Wearing face mask	Always	1,448 (86.4)	394 (96.1)	344 (80.8)	354 (84.3)	356 (84.8)
	Sometimes/never	228 (13.6)	16 (3.9)	82 (19.2)	66 (15.7)	64 (15.2)
Physical distancing	Always	1,187 (70.8)	324 (79.0)	283 (66.4)	265 (63.1)	315 (75.0)
	Sometimes/never	489 (29.2)	86 (21.0)	143 (33.6)	155 (36.9)	105 (25.0)
Cleaning hands with alcohol	≥3 times/day	1,262 (75.3)	389 (94.9)	286 (67.1)	273 (65.0)	314 (74.8)
	< 3 times/day	414 (24.7)	21 (5.1)	140 (32.9)	147 (35.0)	106 (25.2)
Relationships with people around them	Decreased	461 (27.5)	231 (56.3)	100 (23.5)	97 (23.1)	33 (7.9)
	Unchanged/increased	1,215 (72.5)	179 (43.7)	326 (76.5)	323 (76.9)	387 (92.1)

During the fifth wave of the COVID-19 pandemic, 86.4% always wore a face mask, 70.8% always kept a physical distance from others, and 75.3% cleaned their hands with alcohol at least three times each day.

### Changes in agricultural context during the fifth wave of the COVID-19 pandemic

In the context of crop cultivation, the majority of farmers were confronted with the challenge of increased agrochemical costs (77.4%). The highest statistic across all regions was also found to be increased agrochemical costs. The highest proportion was found in the Central region (91.0%), followed by the North (86.2%), the North-East (75.5%), and the South (57.1%).

In the context of crop products, the majority of farmers were presented with the issue of decreased crop product prices (74.9%). The highest impact in the Central was found in decreased crop yields (92.0%), while the highest in the North and the North-East was found in a decrease in crop product prices (84.0 and 66.4%, respectively), and the highest in the Southern region was found in decreased markets of crop products (61.0%).

In the context of economic status, the majority of farmers were confronted with a decrease in household income (82.0%), the greatest impact in the Central area was found to be in increased household expense (96.3%), while the highest impact in the North, the North-East, and the South was found in a decrease in household income (91.5, 80.0, and 69.8%, respectively) (Table 2).

**Table 2:** Changes in agricultural context during the fifth wave of the COVID-19 pandemic in Thailand

Parameter	n (%)				
	**Total (*n*=1676)**	**Central (n=410)**	**North (*n*=426)**	**South (*n*=420)**	**North-East (n=420)**
**Crop cultivation context**					
Increased costs of agrochemicals	1,297 (77.4)	373 (91.0)	367 (86.2)	240 (57.1)	317 (75.5)
Increased costs of crop cultivation	1,167 (69.6)	376 (91.7)	306 (71.8)	176 (41.9)	309 (73.6)
Decreased number of laborers	865 (51.6)	360 (87.8)	244 (57.3)	136 (32.4)	125 (29.8)
Increased difficulty in accessing agrochemicals	793 (47.3)	333 (81.2)	207 (48.6)	166 (39.5)	87 (20.7)
Changed type of agrochemicals	784 (46.8)	358 (87.3)	152 (35.7)	182 (43.3)	92 (21.9)
Decreased agricultural working days	692 (41.3)	300 (73.2)	147 (34.5)	172 (41.0)	73 (17.4)
Changed type of crop cultivation	651 (38.8)	221 (53.9)	169 (39.7)	169 (40.2)	92 (21.9)
Increased quantity of agrochemicals	632 (37.7)	373 (91.0)	121 (28.4)	61 (14.5)	77 (18.3)
Decreased crop protection	608 (36.3)	351 (85.6)	86 (20.2)	144 (34.3)	27 (6.4)
Changed method of crop cultivation	585 (34.9)	240 (58.5)	144 (33.8)	136 (32.4)	65 (15.5)
Decreased rotation of crop cultivation	536 (32.0)	131 (32.0)	179 (42.0)	146 (34.8)	80 (19.0)
Shorter period of crop cultivation	535 (31.9)	248 (60.5)	149 (35.0)	89 (21.2)	49 (11.7)
**Crop product context**					
Decreased prices of crop products	1,255 (74.9)	367 (89.5)	358 (84.0)	251 (59.8)	279 (66.4)
Increased difficulty in accessing markets	1,214 (72.4)	368 (89.8)	347 (81.5)	256 (61.0)	243 (57.9)
Decreased crop yields	1,052 (62.8)	377 (92.0)	271 (63.6)	225 (53.6)	179 (42.6)
Increased difficulty in logistics	1,029 (61.4)	337 (82.2)	315 (73.9)	244 (58.1)	133 (31.7)
**Agricultural economic context**					
Decreased household income	1,374 (82.0)	355 (86.6)	390 (91.5)	293 (69.8)	336 (80.0)
Increased household expense	1,319 (78.7)	395 (96.3)	353 (82.9)	236 (56.2)	335 (79.8)
Increased household debt	994 (59.3)	370 (90.2)	263 (61.7)	179 (42.6)	182 (43.3)
Earning extra income	863 (51.5)	170 (41.5)	287 (67.4)	220 (52.4)	186 (44.3)

### Prevalence of stress, anxiety, and depression during the fifth wave of the COVID-19 pandemic

Stress: 35.1% of farmers had moderate stress, followed by high stress (27.6%), no symptoms (18.4%), mild stress (12.5%), and extremely high stress (6.3%). Considering region, the highest prevalence of extremely high stress was found in the Central (18.3%), followed by the South (12.4%), the North (3.5%), and the North-East (1.4%) regions.

Anxiety: the majority of farmers (61.9%) reported no symptoms, followed by mild symptoms (19.9%), moderate symptoms (12.3%), and severe symptoms (5.9%). Considering region, the highest prevalence of severe symptoms was found in the South (7.4%), followed by the Central (7.1%), the North (5.2%), and the North-East (4.0%).

**Fig. 2:**
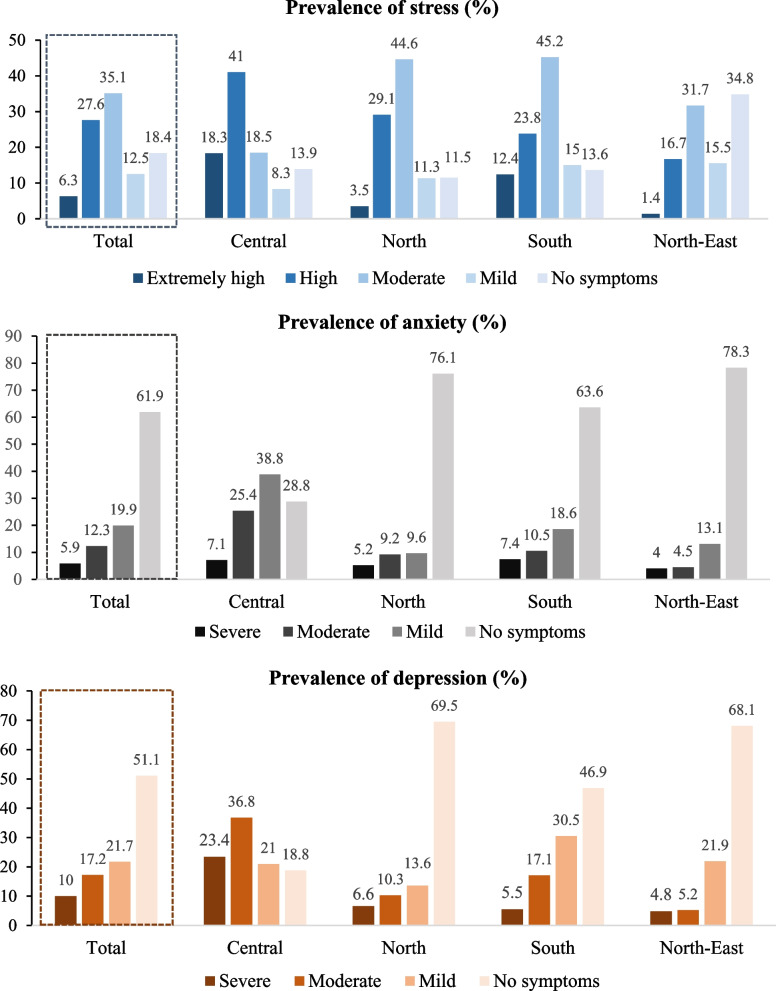
Prevalence of stress, anxiety, and depression among farmers in different regions of Thailand

Depression: Half of the farmers (51.1%) had no symptoms, followed by mild symptoms (21.7%), moderate symptoms (17.2%), and severe symptoms (10.0%). Considering region, the highest prevalence of severe symptoms was found in the Central (23.4%), followed by the North (6.6%), the South (5.5%), and the North-East (4.8%) (Fig. 2).

### Determinants of stress, anxiety, and depression due to changes in agricultural context

Results from the univariate analysis for determinants of mental health of Thai Farmers classified by socio-demographic characteristics are presented in Table 3. Sociodemographic factors associated with the stress levels of farmers were region, monthly income, wearing a face mask, cleaning hands with alcohol, and relationships with people around them. The factors associated with farmer anxiety levels were region, gender, age, marital status, education level, cleaning hands with alcohol, and relationships with people around them. The factors associated with farmer depression levels were region, monthly income, wearing a face mask, cleaning hands with alcohol, and relationships with people around them.

**Table 3:** Univariate analysis for determinants of mental health of Thai Farmers classified by sociodemographic characteristics

Parameter		Stress		Anxiety		Depression	
		**cOR**	**95%CI**	**cOR**	**95%CI**	**cOR**	**95%CI**
Region	Central	3.30	2.41,4.79**	8.95	6.53,12.27**	9.23	6.69,12.73**
	North	4.10	2.60,5.87**	1.14	0.83,1.57	0.94	0.70,1.25
	South	3.39	2.41,4.79**	2.07	1.53,2.81**	2.42	1.83,3.12**
	North-East (ref.)						
Gender	Female	0.912	0.71,1.17	1.40	1.15,1.71**	1.34	1.11,1.63**
	Male (ref.)						
Age	≤ 45 years	1.02	0.79,1.30	2.76	2.25,3.38**	2.83	2.32,3.44**
	> 45 years (ref.)						
Marital status	Single/divorced	1.22	0.93,1.59	1.66	1.35,2.04**	1.58	1.29,1.94**
	Married (ref.)						
Monthly income	THB ≤ 5,000	1.46	1.14,1.87**	0.76	0.62,0.93**	0.69	0.57,0.83**
	THB > 5,000 (ref.)						
Education level	≥ Secondary education	1.27	0.99,1.63	2.07	1.69,2.54**	2.13	1.75,2.59**
	≤ Primary education (ref.)						
Comorbidity	Yes	0.95	0.72,1.25	0.87	0.69,1.09	0.99	0.80,1.23
	No (ref.)						
COVID-19 vaccination	No	1.62	0.87,3.01	0.73	0.47,1.13	0.64	0.42,0.97*
	Yes (ref.)						
Wearing a face mask	Always	1.41	1.01,1.97*	0.95	0.72,1.27	1.05	0.79,1.39
	Sometimes/never (ref.)						
Physical distancing	Always	1.23	0.94,1.59	0.95	0.77,1.19	1.00	0.81,1.24
	Sometimes/never (ref.)						
Cleaning hands with alcohol	≥3 times/day	1.79	1.38,2.35**	1.79	1.40,2.27**	1.65	1.32,2.07**
	< 3 times/day (ref.)						
Relationships with people around them	Decreased	2.99	1.65,3.17**	3.47	2.78,4.34**	3.20	2.55,4.02**
	Unchanged/Increased (ref.)						

*cOR* crude odds ratio, *95%CI* 95% confidence interval

^*^ *P* value < 0.05

^**^ *P* value < 0.01

Results from the univariate analysis for determinants of mental health of Thai Farmers classified by changes in agricultural context are presented in Table 4. The factors associated with the mental health of the farmers were as follows: type of crop cultivation, crop rotation, duration of crop cultivation, method of crop cultivation, agricultural working days, number of laborers, crop protection, costs of crop cultivation, access to agrochemicals, quantity of agrochemicals, type of agrochemicals, crop yields, prices of crop products, markets of crop products, logistics of crop products, and household debt.

**Table 4:** Univariate analysis for determinants of mental health of Thai Farmers classified by changes in agricultural context

Parameter	Stress		Anxiety		Depression	
	**cOR**	**95%CI**	**cOR**	**95%CI**	**cOR**	**95%CI**
**Crop cultivation context**						
Changed type of crop cultivation	2.64	1.97,3.52**	1.61	1.32,1.97**	1.77	1.45,2.16**
Decreased rotation of crop cultivation	2.71	1.97,3.72**	1.48	1.19,1.82**	1.83	1.49,2.26**
Shorter period of crop cultivation	1.97	1.46,2.64**	2.29	1.86,2.83**	2.49	2.01,3.08**
Changed method of crop cultivation	2.25	1.68,3.02**	2.17	1.77,2.67**	2.39	1.95,2.95**
Decreased agricultural working days	3.49	2.58,4.70**	2.94	2.39,3.61**	3.71	3.02,4.56**
Decreased number of laborers	1.89	1.47,2.43**	2.46	2.01,3.01**	2.37	1.95,2.88**
Decreased crop protection	2.17	1.63,2.89**	3.89	3.15,4.81**	4.63	3.73,5.75**
Increased costs of crop cultivation	0.78	0.59,1.03	1.32	1.06,1.64*	0.83	0.68,1.03
Increased difficulty in accessing agrochemicals	2.15	1.66,2.79**	2.40	1.96,2.94**	2.22	1.82,2.69**
Increased quantity of agrochemicals	1.76	1.34,2.31**	3.69	2.99,4.55**	3.34	2.71,4.11**
Changed type of agrochemicals	2.51	1.92,3.28**	3.61	2.93,4.44**	3.85	3.14,4.71**
Increased costs of agrochemicals	0.89	0.66,1.21	0.96	0.76,1.22	0.91	0.73,1.15
**Crop product context**						
Decreased crop yields	2.54	1.98,3.27**	1.95	1.58,2.41**	2.07	1.69,2.54
Decreased prices of crop products	1.65	1.26,2.16**	1.50	1.19,1.89**	1.43	1.14,1.79**
Increased difficulty in accessing markets	1.19	0.91,1.56	1.60	1.27,2.01**	1.53	1.23,1.89**
Increased difficulty in logistics	1.63	1.27,2.09**	1.86	1.50,2.29**	1.69	1.38,2.06**
**Agricultural economic context**						
Decreased household income	0.93	0.67,1.29	1.11	0.86,1.44	0.89	0.69,1.14
Increased household expense	1.05	0.78,1.42	1.06	0.83,1.35	0.94	0.74,1.19
Increased household debt	2.11	1.64,2.71**	2.65	2.14,3.28**	2.75	2.25,3.37**
Earning extra income	1.09	0.85,1.39	0.89	0.74,1.09	0.85	0.70,1.03

*cOR* crude odds ratio, *95%CI* 95% confidence interval

^**^ *P* value < 0.01

The results of the multivariate analysis for determining the factors associated with the mental health of farmers due to changes in agricultural context after adjusting covariates are presented in Table 5. The results found that agricultural factors associated with stress were changed crop cultivation type (adj.OR= 1.59, 95%CI=1.09,2.32), decreased number of agricultural working days (adj.OR=2.54, 95%CI=1.69,3.83), increased difficulty in accessing agrochemicals (adj.OR=1.45, 95%CI=1.03,2.04), changed agrochemical types (adj.OR=1.52, 95%CI=1.05,2.19), decreased crop yields (adj.OR=1.45, 95%CI=1.04,2.04), increased difficulty in accessing markets (adj.OR=1.79, 95%CI=1.24,2.58), and increased household debt (adj.OR=1.56, 95%CI=1.12,2.17).

**Table 5:** Multivariate analysis for determinants of mental health of Thai farmers due to changes in agricultural context during the fifth wave of the COVID-19 pandemic

Determinants	Stress ^a^		Depression ^b^		Anxiety ^c^	
	**aOR**	**95%CI**	**aOR**	**95%CI**	**aOR**	**95%CI**
**Crop cultivation context**						
Change in type of crop cultivation	1.59	1.09,2.32*	0.76	0.57,1.02	0.77	0.57,1.03
Decreased crop rotation	1.33	0.89,1.98	1.65	1.22,2.23**	1.36	1.01,1.84*
Decreased crop duration	0.75	0.49,1.16	1.03	0.74,1.43	0.99	0.72,1.37
Changed method of crop cultivation	0.95	0.62,1.46	0.99	0.71,1.36	1.03	0.75,1.43
Decreased agricultural working days	2.54	1.69,3.83**	1.83	1.36,2.46**	1.39	1.03,1.88*
Decreased labors	0.84	0.59,1.21	0.89	0.66,1.19	1.04	0.77,1.41
Decreased crop protection	0.79	0.48,1.28	1.27	0.90,1.77	1.04	0.74,1.46
Increased difficulty in accessing agrochemicals	1.45	1.03,2.04*	0.89	0.69,1.18	0.98	0.75,1.29
Increased quantity of agrochemicals	0.97	0.65,1.45	1.13	0.82,1.54	1.29	0.94,1.77
Changed agrochemical types	1.52	1.05,2.19*	1.73	1.32,2.29**	1.59	1.20,2.12**
**Crop products context**						
Decreased crop yields	1.45	1.04,2.04*	0.75	0.56,1.01	0.69	0.52,1.95
Decreased crop product prices	1.21	0.86,1.69	0.97	0.72,1.30	0.98	0.72,1.33
Increased difficulty in accessing markets	1.79	1.24,2.58**	1.14	0.83,1.57	1.17	0.83,1.63
Increased difficulty in logistics	0.79	0.55,1.13	0.90	0.67,1.22	1.05	0.77,1.42
**Agricultural economic context**						
Increased household debt	1.56	1.12,2.17**	1.75	1.34,2.28**	1.45	1.09,1.90**

*aOR* adjusted odds ratio, *95%CI* 95% confidence interval

^*^ *P *value < 0.05

^**^ *P* value < 0.01

^a^ adjusted with region, monthly income, wearing face mask, cleaning hands with alcohol, and relationship with people around

^b^ adjusted with region, gender, age, marital status, monthly income, education level, COVID-19 vaccination, cleaning hands with alcohol, and relationship with people around

^c^ adjusted with region, gender, age, marital status, monthly income, education level, cleaning hands with alcohol, and relationship with people around

As regards anxiety and depression of farmers, the associated factors included decreased crop rotation (adj.OR=1.65, 95%CI=1.22,2.23 for anxiety and adj.OR=1.36, 95%CI=1.01,1.84 for depression), decreased number of agricultural working days (adj.OR=1.83, 95%CI=1.36,2.46 for anxiety and adj.OR=1.39, 95%CI=1.03,1.88 for depression), changed agrochemical types (adj.OR=1.73, 95%CI=1.32,2.29 for anxiety and adj.OR=1.59, 95%CI=1.20,2.12 for depression), and increased household debt (adj.OR=1.75, 95%CI=1.34,2.28 for anxiety and adj.OR=1.45, 95%CI=1.09,1.90 for depression).

## Discussion

The COVID-19 pandemic had a significant impact on the agricultural sector worldwide especially as regards food production, distribution, consumption, and food security. In Thailand, the agricultural sector is primarily located in rural areas and employs around 35% of Thailand's workers. Therefore, this sector is a key part of the Thai economy [23].Our results suggested that the fifth wave of the COVID-19 pandemic in Thailand was associated with changes in agricultural sectors in terms of crop cultivation, crop products, and economic status. The majority of Thai farmers faced the problems of increased costs of agrochemicals, decreased logistics as regards crop products, and a decrease in household income during the fifth wave of the COVID-19 pandemic. The findings were in agreement with the study by Wasito et al. [24] which clearly showed that the COVID-19 pandemic resulted in a 5% decline in agricultural yields due to logistic problems during country lockdown measures. The logistical problems during the country lockdown measures also led to greater difficulty in accessing agricultural input supplies. This resulted in higher prices of seeds, agrochemicals, and fertilizers [25]. In addition, border restriction measures also led to a shortage of agricultural inputs and migrant laborers [22]. These factors all resulted in a reduction in household income and agricultural households drew on their savings resulting in an increase in their debt to compensate for their lower monthly income [18].

Lockdown and physical distancing measures were crucial factors in resulting in a decrease in agricultural working days. Agricultural markets and consumer behavior were also affected by these measures. Consumer purchasing power and demand were diminished due to income loss of consumers. These all resulted in the reduction of crop cultivation, crop rotation, and agricultural working days as a result of decreased market demand. Consequently, farmers lost income and incurred debt during the pandemic [26].The Thai government provided relief measures during the pandemic offering compensation for informal workers of 5,000 Thai baht for three months. However, this money needed to be transferred through a bank account registered with PromtPay by ID card and the majority of farmers do not have access to the internet and are illiterate [27]. As a result, most farmers found it impossible to obtain compensation under this approach and the relief measure needs to be expanded to enable informal workers in agriculture and rural sectors to access this help [25].

Considering the impact of the COVID-19 pandemic by region, farmers in the Central region had the highest to confront the changes in agricultural context, followed by the North, the South, and the North-East, respectively. In addition, farmers in the Central region had the highest prevalence of mental health. It is possible that difference in income before and after the country lockdown measures. The study by Komin et al. [18] clearly stated that the percentage change in income before and after the lockdown measures was 91% for the Central, 81% for the North-East, 80% for the North, and 69% for the South. Therefore, specific measures and policies from government and regional institutions should be tailored toward a target region to solve the problems pertinent to that area.

The COVID-19 pandemic has had significant psychological impact on farmers. Our results found that 87.5% of Thai farmers were stressed, 38.1% were anxious, and 48.9% had depressive symptoms during the fifth wave of the COVID-19 pandemic. In comparison, the findings of Sapbamrer et al. [9], who determines the effects on mental health among northern Thai farmers during the fourth wave of the COVID-19 pandemic, indicated that 98.1% of northern Thai farmers were stressed and 19.6% showed depressive symptoms. Therefore, Thai farmers were likely to have less stress but more symptoms of depression during the fifth wave of the COVID-19 pandemic. Although measures given by the Thai government in the fifth wave were more relaxed than in the fourth wave, long periods of the measures could result in the development of severe mental health.Comparing with the global mental health during the COVID-19 pandemic, the prevalence of stress, anxiety, depression, and psychological distress was 36.5, 26.9, 28, and 50%, respectively [14]. Therefore, the evidence showed that the prevalence of mental health in Thai farmers had higher than those in global population.

Psychological stress is a physical and biological process to fight-or-flight response during a situation. Healthy and young people may be able to adapt to the stress response and there is a lower health burden. However, people who experience persistent stress, which they feel is unmanageable, can develop anxiety and depression [28, 29].Anxiety is an emotion characterized by tension, worried thoughts, fear, apprehension, and physical changes. It can lead to excessive nervousness, fear, apprehension, and worry [30]. Depression is a significant mood disease that affects how people feel, think, and handle their everyday lives. It leads to sadness and a loss of interest in activities, as well as a variety of mental and physical problems [31]. Our findings suggested that the fifth wave of COVID-19 in Thailand caused farmers to be more anxious and depressed.

Interestingly, the significant findings were that the crucial agricultural factors associated with farmers’ mental health were decreased number of agricultural working days, changed agrochemical types, changed crop cultivation type, decreased crop rotation, increased difficulty in accessing agrochemicals and markets, decreased crop yields, and increased household debt. These results agreed with previous studies [9, 15]. Lockdown and physical distancing measures threatened crop production and crop yields. These measures also limited access to farms and agrochemical availability, resulting in reduced crop yields. Finally, as a result of these circumstances, farmers’ debt rose [9, 15, 32]. The study by Shin et al. [33] clearly showed that financial problems were the most common cause of stress which led to depression and suicidal ideation. In order to reduce the negative impact on the mental health of farmers, governmental and relevant institutions should provide supportive measures and facilities for facilitating agricultural practices in terms of crop cultivation, crop products, and agricultural economic status. They need to facilitate agricultural inputs and make markets more accessible and keep pricing in check. Improvement of the logistical systems should be considered in order to deliver products to warehouses and customers efficiently [15]. The study by Bright et al. [34] suggested that online marketing is a strategy for improving markets and food supply during the pandemic which could also be helpful in Thailand. All these supportive measures could increase the number of agricultural working days and prevent the cessation of crop cultivation of farmers. Critically, improvements of the mental health care system for more accessible and high-quality mental health services should be carried out to reduce the long-term psychological effects of the COVID-19 pandemic [35, 36].

This data collection during the fifth wave of COVID-19 pandemic in all regions of Thailand will enable the government and researchers to gain increased awareness about the current situations of the impact of COVID-19. However, there are some limitations to this study. This study focused on the agricultural factors associated with the mental health of farmers. However, some social aspects, such as norms, social inequality, and culture, might be affected by mental health. Further research on social factors is needed to be considered. This study focused only on the impact of COVID-19 over a short time, in effect a snapshot at the start of the fifth wave. Furthermore, a cross-sectional study may not establish a cause-and-effect relationship. A follow-up survey and further research are needed for more continuous monitoring of the long-term effects of the COVID-19 pandemic.

## Conclusion

The findings indicate that the majority of Thai farmers faced problems of increased cost of agrochemicals, decreased logistics, and a decrease in household income during the fifth wave of the COVID-19 pandemic. Farmers in the Central region experienced the highest impact with regard to the changes in agricultural context, and had the highest prevalence of mental health problems. One significant finding was that the crucial agricultural factors associated with the mental health of farmers were decreased number of agricultural working days, changed agrochemical type, changed crop cultivation type, decreased crop rotation, increased difficulty in accessing agrochemicals and markets, decreased crop yields, and increased household debt.

The findings of this study will be useful for the government and other institutions to provide supportive measures and facilities, as well as improve the mental health of farmers during and after the COVID-19 pandemic suit on the regions. Specific measures and facilities should be tailored towards the target regions. Technical measures both in the short- and long-term are required to support agricultural infrastructure and agricultural input supply, improving logistics, and controlling market forces through pricing mechanisms. Controlling of the prices of agrochemicals, fertilizers, and seeds is also required, with additional economic support measures. Government also need to provide direct financial compensation and subsidies for vulnerable farmers with easy access to these supporting packages [10, 32, 37]. Short-term loan programs may be required to support small agricultural businesses in rural areas. More effort is needed to maintain agricultural supply chains and strengthen the market linkages for local producers [25, 32].

Improvement in the mental health care system with greater availability and high-quality mental health services should be accessible for reducing long-term psychological effects of the COVID-19 pandemic [35, 36]. Adoption of innovation as regards online services and other forms of virtual care (such as telepsychiatry and health applications) could be used to screen and diagnose the farmers with mental health problems in a timely manner and reduce severe cases of mental health illness [35, 38, 39]. Primary health care plays an important role in the prevention of diseases and the promotion of the health of people in communities, especially in rural areas [40]. Health promoting hospitals in all subdistricts of Thailand acts as primary health care services in Thailand, therefore, enhancing the capacity of health promoting hospitals and integrating mental healthcare into these institutions should be considered to facilitate the monitoring and screening of the mental health of vulnerable people and farmers in particular. All supportive measures should be implemented to mitigate the short and long-term impact of COVID-19 on the agricultural system and the mental health of farmers.

## Supplementary Information

**Additional file 1: Figure S1. **The theoretical framework of the study.

## Data Availability

The data used in the study can be made available from Ratana Sapbamrer (corresponding author) on reasonable request.

## Abbreviations

aOR: adjusted odds ratio
ATK: antigen test kit
COVID: corona virus disease
cOR: crude odds ratio
GDP: total domestic product
%: percentage
95%CI: 95% confidence interval
*: *P* value < 0.05
**: *P* value < 0.01
