# Supplementary figures and images for: Changes in agricultural context and mental health of farmers in different regions of Thailand during the fifth wave of the COVID-19 pandemic
Source: BMC Public Health. 2022 Nov 9;22:2050. doi: 10.1186/s12889-022-14464-3 (PMC9644387; doi:10.1186/s12889-022-14464-3)

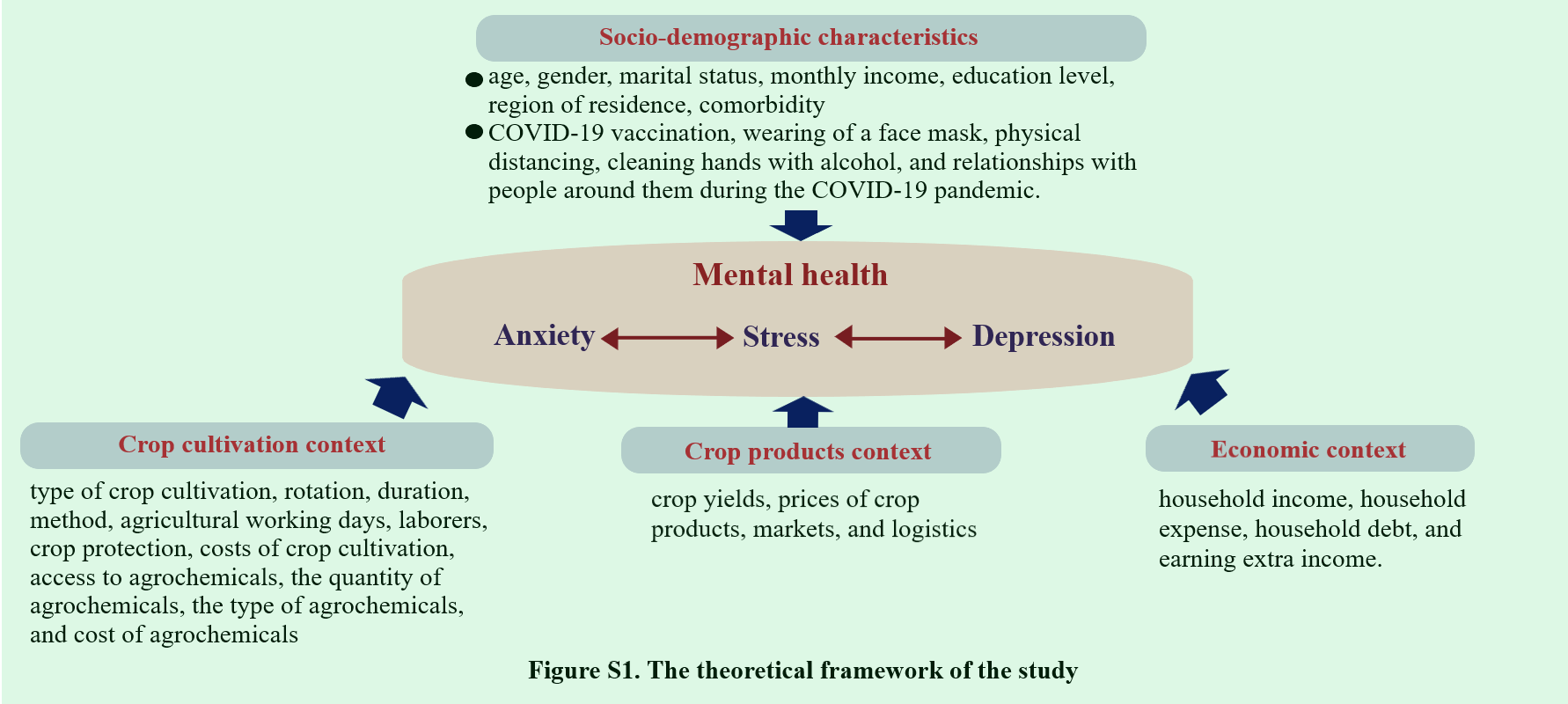

Supplement: Supplementary file 1 — Additional file 1: Figure S1. The theoretical framework of the study. [file 12889_2022_14464_MOESM1_ESM.tiff]
